# Editorial: The role of cannabinoids and the endocannabinoid system in anti-cancer therapy

**DOI:** 10.3389/fphar.2026.1754350

**Published:** 2026-01-27

**Authors:** Lirit N. Franks, Wesley Raup-Konsavage, Bruno M. Fonseca

**Affiliations:** 1 Division of Epidemiology, Department of Internal Medicine, University of Utah School of Medicine, Salt Lake City, UT, United States; 2 Department of Neuroscience and Experimental Therapeutics, The Pennsylvania State University College of Medicine, Hershey, PA, United States; 3 Applied Molecular Biosciences Unit (UCIBIO) and Associate Laboratory i4HB, Biochemistry Laboratory, Faculty of Pharmacy, University of Porto, Porto, Portugal

**Keywords:** anti-tumor mechanisms, cancer therapy, cannabinoids, chemotherapy synergy, endocannabinoid system, palliative care

Cancer is an increasing global health problem with a rising 2.3 fold incidence over the last 30 years and, currently, 1 in 5 people will develop cancer in their lifetime. (Zhu et al., 2025; Bray et al., 2024). Due to the variability in effectiveness of traditional treatments, there is a perpetual need to find new or improved therapies. Many patients turn to holistic and plant medicine to improve their quality of life and in hope of increasing the success of traditional chemotherapeutics. (Aycil and Karaca, 2025). Among plant medicines, one of the most common is the use of cannabis. (Martin et al., 2025; Hardy et al., 2025). Many patients use cannabis for palliative care, symptom management or to combat adverse reactions from cancer treatments. (Hardy et al., 2025). There are a few cannabinoid medications currently approved by drug agencies to help manage adverse effects caused by cancer chemotherapy. Synthetic Δ^9^-THC (dronabinol) and a synthetic analogue, nabilone, are approved for the treatment of nausea, vomiting, and lack of appetite in the United States and most of Europe. (Health Information, 2025). Cannabis-based medicinal products are now officially approved in several countries, including Portugal, Germany, and Italy, for the management of cancer-related symptoms such as pain, nausea and cachexia (Correa and Tucci, 2025).

Some patients, however, hope to battle the tumor itself using cannabis or cannabinoids, and there is some pre-clinical evidence for this intent. In many cancers, there is a dyregulation of phytocannabinoid receptors, including the CB_1_ and CB_2_ receptors and GPR55. (Perez-Gomez et al., 2015; Song et al., 2023; Shoeib et al., 2021; Wnorowski et al., 2021). Targeting these receptors and/or altering endocannabinoids has shown promise in pre-clinical trials. (Velasco et al., 2012; Lee et al., 2022; Elbaz et al., 2017; Oh et al., 2013). Some cannabinoids have been found to inhibit tumor progression or induce apoptosis in various cancer cell lines. (Cianchi et al., 2008; Fonseca et al., 2018; Patsos et al., 2010; Sarfaraz et al., 2006). Other studies suggest that cannabinoids exhibit anti-angiogenic properties, disrupt signaling pathways that promote tumor proliferation thus impeding tumor progression, or modulate the immune system to foster an anti-tumor response. (Elbaz et al., 2017; Ramer et al., 2022; Casanova et al., 2003; Kogan et al., 2006; Pisanti et al., 2011; Portella et al., 2003; Ravi et al., 2016; Braile et al., 2021; Dada et al., 2022). There is also evidence that cannabinoids in conjunction with chemotherapeutics can provide improved therapy outcomes in humans, particularly with glioblastoma. (Kyriakou et al., 2021; Bhaskaran et al., 2024). Alternatively, there are studies that suggest that cannabinoids can promote cancer proliferation and could be contraindicated for certain cancers or interfere with existing therapies. (Li et al., 2021; Hasenoehrl et al., 2018; Hu et al., 2011; Martinez-Martinez et al., 2016).

Cannabis use is common among cancer patients and, due to the heterogeneity of cancers and cannabis products being used, there is inconclusive evidence on how cannabis can help or hurt. The aim or our Topic was to highlight recent research on the role of cannabis, cannabinoids, or the endocannabinoid system with regards to treating cancer.

To understand the larger picture of publication frequency and topics for cannabinoids/endocannabinoid system and cancer, Tan et al. conducted a bibliometric analysis of the current literature. They found that from 1995–2024, 3,052 publications were identified using the Web of Science Core Research Topic database spanning manuscripts from 86 countries and 3,362 institutions. The majority of publications on the topic were published after 2021. While most the top 100 publishing institutions were in the United States, cannabis and cancer research was most frequently done in Italy and Spain, with those countries hosting researchers that have published on the topic most frequently. The authors analyzed frequent keywords and revealed several “hotspots” including treatment for cancer-symptoms, (particularly nausea, vomiting, and pain) and the growing evidence for anti-tumor effects of cannabinoid (mostly Δ^9^-THC and CBD).

An overview of the therapeutic potential of the endocannabinoid system in cancer treatment is presented in a mini-review by Salum et al. The authors explain the endocannabinoid system, how it regulates physiological processes, and how these can potentially contribute to anticancer effects. This is followed by a summary of global impact of cancer, overview of carcinogenesis, and how the endocannabinoid system could interconnect. Specific studies of cannabinoids that suggest anti-tumor effects, including inhibition of tumor growth, cytotoxic effects, and modulation of angiogenesis, are presented. They also caution that some cannabinoids have pro-tumor effects based on context and tumor heterogeneity.

On a more granular level, Ravnik et al. explored possible molecular targets for phytocannabinoids by using an inverse molecular docking approach to search for potential protein targets, many of which are associated with cancer. Cannabinoids demonstrated protein binding similarity in two main categories 1) CBD, CBC, and CBG and 2) Δ^9^-THC and similar, including CBL-class, presenting opportunities for therapeutic benefits associated with THC but with less psychotropic effects. Two main targets were associated with more favorable docking scores with 12 out of 14 cannabinoids tested: 1) GTPase KRas, a regulator of cell survival, differentiation and growth and which has not been historically receptive to pharmacotherapy attempts and 2) hematopoietic cell kinases, which are involved in cellular homeostasis, innate immune response, and have been linked to the onset of various cancers.

Another angle for therapeutic development of cannabinoids was approached by Vigano et al. by reviewing the evidence of cannabinoids’ ability to modulate the pharmacodynamics of immunotherapy checkpoint inhibitors. They detail the concept of immune checkpoints for inhibitor immunotherapy during cancer treatment, receptors associated with the endocannabinoid system and their interaction with the immune system. In discussing specific studies, the authors highlight that cannabinoids have been shown to inhibit T-cell mediated response in murine models. Observational studies found cannabinoid use leads to decreased time for tumor progression but minimized adverse events associated with immune therapy. The authors caution that studies to date are not uniform and need more statistical power.

Two *in vitro* studies delved into the mechanisms by which cannabinoids could affect cancer cells. Tong et al. conducted an *in vitro* study with two lines of ovarian cancer cells (SKOV3 and A2780) assessing the cytotoxicity of CBD, Δ^9^-THC, and both combined. The authors demonstrated cytotoxicity of both compounds in cancer cells lines, without harming the non-tumor IOSE cells, with ratio- and concentration-dependent synergy. The combination more effectively inhibited proliferation, impaired cellular migration, and induced apoptosis by down-regulating phosphorylation of and thus suppressing the PI3L/AKT/mTOR signaling pathway while restoring the tumor suppression function of PTEN.

Chen at al. explored the *in vitro* cytotoxic effects of natural cannabinoids and their synthetic derivatives in human ovarian cancer cells. CBD and CBN exhibited the greatest cytotoxicity with CBD showing more antiproliferative effects. The authors created fifty-six synthetic analogs of CBD and two of those showed enhanced cytotoxic effects. Both analogs contained an added piperazine moiety. At IC_50_ concentrations, these two analogs did not show apoptotic effects but did show enhanced iron levels for ferroptosis, although mechanism of cell death still needs to be confirmed. They assessed these analogs for synergistic effects with cisplatin, and one analog enhanced the antiproliferative effects of cisplatin. This is of particular interest in developing medications for chemotherapy-resistant ovarian cancers.

In conclusion, it is evident that research about cannabinoids and the endocannabinoid affecting cancer treatment is on the rise. *In vitro* studies continue to show promise for cannabinoids to enhance treatments. Targets are expanding beyond direct cytotoxic effects to include synergistic combinations with established therapies and the exploration of novel molecular targets, supporting an integrative therapeutic role now being explored in multiple clinical trials. Continued research on how cannabinoids can assist with anti-tumor treatment both by enhancing anti-tumor treatments and improving patients’ quality of life is still essential. Encouraging evidence is steadily growing, providing strong momentum toward clinical applications.
